# Supplementary material for: A Multi-tissue Transcriptome Analysis of Human Metabolites Guides Interpretability of Associations Based on Multi-SNP Models for Gene Expression
Source: Am J Hum Genet. 2020 Jan 23;106(2):188–201. doi: 10.1016/j.ajhg.2020.01.003 (PMC7010967; doi:10.1016/j.ajhg.2020.01.003)
Supplement: Document S1. Figures S1 and S2 [file mmc1.pdf]

The American Journal of Human Genetics, Volume *106*

## Supplemental Data

# A Multi-tissue Transcriptome Analysis of Human Metabolites Guides Interpretability of Associations Based on Multi-SNP Models for Gene Expression

**Anne Ndungu, Anthony Payne, Jason M. Torres, Martijn van de Bunt, and Mark I. McCarthy**

## SUPPLEMENTAL FIGURES

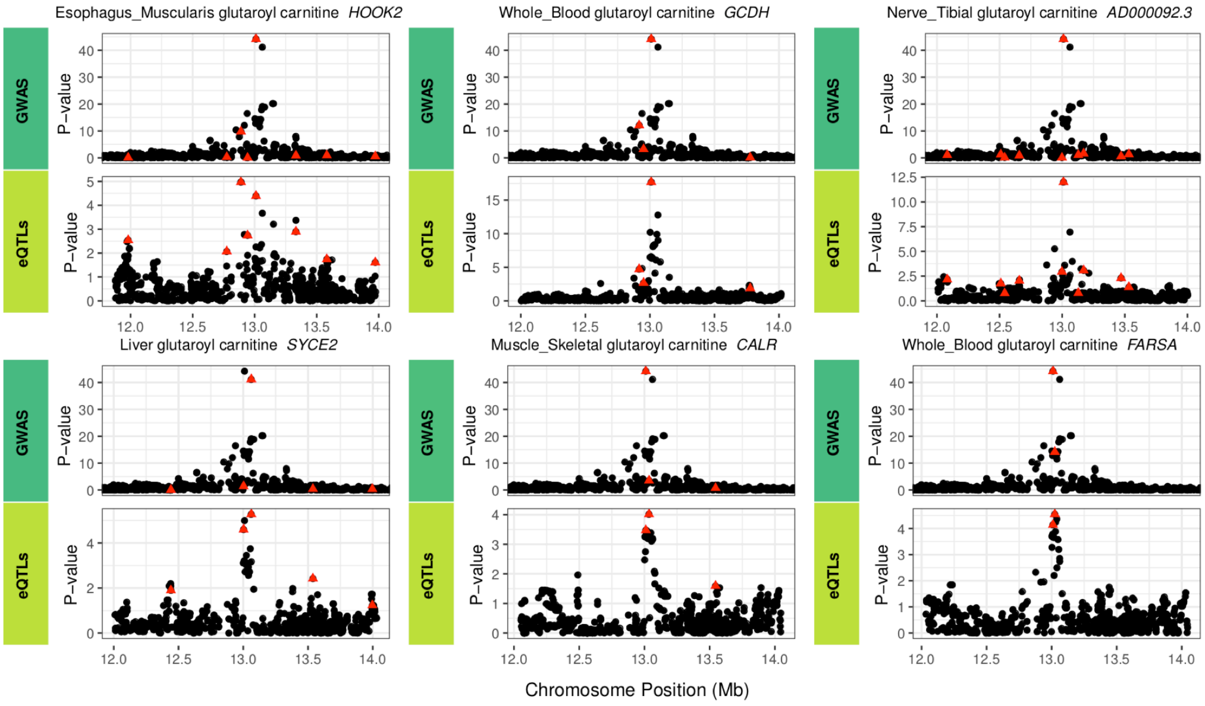


**Figure S1. Gene expression co-regulation at the *GCDH* locus.** Six genes at the *GCDH* locus whose predicted expression significantly mediated glutarylcarnitine levels. The multi-SNP models for all six genes included the *cis*-eQTL rs8012 that regulates the expression for all the genes. *GCDH* is the true causal gene influencing metabolite levels at this locus.

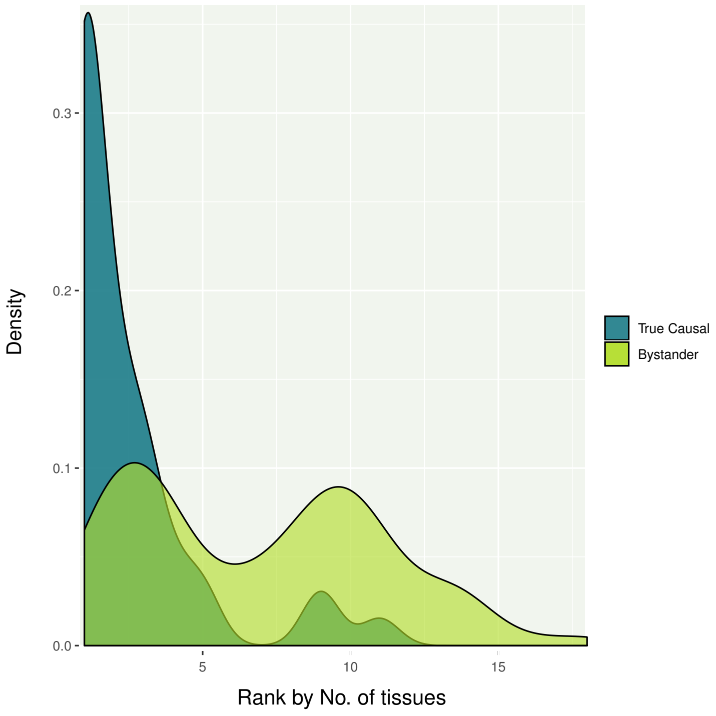


**Figure S2. Causal and bystander gene ranks by number of tissues**. Comparison of ranks by tissue counts for true causal genes and bystander genes at each locus. True causal genes ranked higher (significant in a more tissues) than bystander genes at the same locus.
