# Supplementary material for: A Multi-tissue Transcriptome Analysis of Human Metabolites Guides Interpretability of Associations Based on Multi-SNP Models for Gene Expression
Source: Am J Hum Genet. 2020 Jan 23;106(2):188–201. doi: 10.1016/j.ajhg.2020.01.003 (PMC7010967; doi:10.1016/j.ajhg.2020.01.003)
Supplement: Document S3. Article plus Supplemental Information [file mmc3.pdf]

ARTICLE

A Multi-tissue Transcriptome Analysis of Human Metabolites Guides Interpretability of Associations Based on Multi-SNP Models for Gene Expression

Anne Ndungu,[1,5] Anthony Payne,[1,5] Jason M. Torres,[1,5] Martijn van de Bunt,[1,2,3,6] and Mark I. McCarthy[1,2,4,6,*]

There is particular interest in transcriptome-wide association studies (TWAS) gene-level tests based on multi-SNP predictive models of gene expression—for identifying causal genes at loci associated with complex traits. However, interpretation of TWAS associations may be complicated by divergent effects of model SNPs on phenotype and gene expression. We developed an iterative modeling scheme for obtaining multi-SNP models of gene expression and applied this framework to generate expression models for 43 human tissues from the Genotype-Tissue Expression (GTEx) Project. We characterized the performance of single- and multi-SNP models for identifying causal genes in GWAS data for 46 circulating metabolites. We show that: (A) multi-SNP models captured more variation in expression than did the top *cis*-eQTL (median 2-fold improvement); (B) predicted expression based on multi-SNP models was associated (false discovery rate < 0.01) with metabolite levels for 826 unique gene-metabolite pairs, but, after stepwise conditional analyses, 90% were dominated by a single eQTL SNP; (C) among the 35% of associations where a SNP in the expression model was a significant *cis*-eQTL and metabolomic-QTL (met-QTL), 92% demonstrated colocalization between these signals, but interpretation was often complicated by incomplete overlap of QTLs in multi-SNP models; and (D) using a "truth" set of causal genes at 61 met-QTLs, the sensitivity was high (67%), but the positive predictive value was low, as only 8% of TWAS associations (19% when restricted to colocalized associations at met-QTLs) involved true causal genes. These results guide the interpretation of TWAS and highlight the need for corroborative data to provide confident assignment of causality.

Introduction

Genome-wide association studies (GWAS) have been powerful tools in revealing many loci that influence complex traits and diseases. However, most SNP associations map to non-coding regions of the genome, thereby complicating the task of identifying the (causal) genes through which the observed effects on disease predisposition are mediated.[1] To address this challenge, researchers have implemented a variety of approaches to link regulatory variants implicated in disease predisposition to their downstream effectors. One of the most widely adopted approaches leverages expression quantitative trait loci (eQTLs) to identify regional genes that are under the direct regulatory influence of the disease risk variant(s) and which thereby represent candidate mediators of disease predisposition. Empirical support for this approach is provided by the enrichment of *cis*-eQTL regulatory variants among significant GWAS variants and evidence that such variants explain a disproportionate share of trait heritability.[2–6]

A range of approaches have been deployed to detect coincident *cis*-eQTL and trait association signals. The simplest involves limiting the search space to trait variants that also demonstrate significant eQTL signals in a disease-relevant tissue. In such analyses, it is now widely accepted that it is essential to test for statistical evidence of colocalization between eQTLs and trait-associated SNPs in order to avoid assigning relationships between eQTL and trait signals that map to distinct causal variants, and which cannot therefore be assumed to have any biological connection.[7,8]

Recently, this approach has been supplemented by a suite of methods (collectively, transcriptome-wide association studies [TWAS]), built around a Mendelian randomization (MR) framework, which test for relationships between the genetic components of both complex traits and gene expression.[5,9–13] For example, the PrediXcan method generates predictive models of transcript expression from eQTL mapping data, and then uses these to "impute" estimates of gene expression into case-control or cohort-based GWAS datasets; those imputed estimates can then be subjected to trait association testing.[12] Although PrediXcan requires individual-level genotype data as input, conceptually similar approaches are available that can accept GWAS summary statistics with linkage disequilibrium (LD) estimates from a reference population (e.g., S-PrediXcan, Fusion).[5,13,14] Collectively, these methods have been applied to a broad range of complex traits and diseases and have spotlighted previously unreported and biologically plausible disease

[1]The Wellcome Centre for Human Genetics, Nuffield Department of Medicine, University of Oxford, Oxford, OX3 7BN, UK; [2]Oxford Centre for Diabetes, Endocrinology and Metabolism, Radcliffe Department of Medicine, University of Oxford, Oxford, OX3 7LE, UK; [3]Department of Bioinformatics and Data Mining, Novo Nordisk A/S, Måløv, 2760, DK
[4]Present address: OMNI Human Genetics, Genentech, 1 DNA Way, South San Francisco, CA 94080, USA
[5]These authors contributed equally to this work
[6]These authors contributed equally to this work
*Correspondence: mccarthy.mark@gene.com
https://doi.org/10.1016/j.ajhg.2020.01.003.
© 2020 The Author(s). This is an open access article under the CC BY license (http://creativecommons.org/licenses/by/4.0/).

gene candidates that had evaded detection in conventional GWAS approaches.[5,11–13]

The prediction models generated by these approaches range from those that feature only the single best (i.e., most strongly associated) eQTL for each gene, to those that support a polygenic model which comprises all SNPs within a locus (e.g., best linear unbiased predictor [BLUP]). However, it has been shown that more sparse multivariate linear models (such as those generated by Least Absolute Shrinkage and Selection Operator [LASSO] regression or a bayesian sparse linear mixed model [BSLMM]) outperform both single-variant and polygenic models in predicting gene expression.[5,11–13,15] Unlike single-variant models, these sparse multi-SNP models can capture the effects of allelic heterogeneity (i.e., genes whose transcription is under the influence of multiple *cis*-regulatory signals). They also better reflect current understanding of the genetic architecture of gene expression than do polygenic models.[5,12,15]

The fact that multi-SNP models better predict gene expression than single-SNP models do might suggest that trait associations based on these models would themselves involve multiple SNPs with shared effects on both expression and phenotype. However, the extent to which this is true is unknown. Moreover, if such models better reflect the number of independent genetic signals acting on a phenotype, are they supported by evidence of shared identity between the trait-associated and eQTL variants within the model? Furthermore, the extent to which genes implicated by colocalized associations represent genuine biological relationships, causal for disease, is unclear, and inference is further complicated by the shared regulatory architecture of gene expression and by horizontal pleiotropy.[16,17]

To address these outstanding questions and guide the interpretability of predicted gene expression studies, we systematically evaluated sparse multi-SNP models underlying significant gene associations for evidence of independent effects on both phenotype and expression. We did this by generating multi-SNP gene expression models for 43 human tissues from the GTEx project and evaluating their utility through a large-scale analysis of GWAS data for 46 metabolites. We focused on metabolomic phenotypes because they provide a singular opportunity to assess the biological plausibility of multi-SNP gene associations. Insights from both genetic and experimental studies have led to well-curated sets of effector genes at loci with *cis*-associations to the levels of particular metabolites.[18–21] The subsets of genes so implicated encode enzymes, transporters, and regulators that can be directly tied to the specific metabolite based on known functional relationships. These provide a "truth" gene set that can then be used to assess the performance (i.e., sensitivity and positive predictive value [PPV]) of alternative analytical approaches for identifying effector transcripts, and which can inform the utility of applying TWAS approaches to the interpretation of GWAS data for other complex traits.

Material and Methods

GTEx Expression Data and *Cis*-eQTL Analysis

Genotype data (variant call format), gene expression (quantified gene-level counts), and sample phenotype data from GTEx version 7 were obtained through dbGaP accession number phs000424.v7.p2.[22] Genotypes were filtered to keep only bi-allelic variants with minor allele frequency of at least 0.05. Finally, to ensure consistent downstream modeling and testing across metabolites, we selected 2,539,611 SNPs that overlapped between GTEx genotypes and the HapMap2-imputed metabolite GWAS.

Only non-sex-specific tissue types with a sample size of $n \geq 50$ were analyzed. For each tissue, genes reaching a threshold of >6 raw reads and >1 count per million in at least 10 individuals were carried forward for analysis. Remaining genes were normalized using the trimmed mean of M-values (TMM), then log transformed to counts per million through the use of the Voom function in the R package limma.[23] To account for hidden systematic confounders in the data, we calculated surrogate variables for each tissue, after explicitly accounting for sex, using the R package sva (version 3.22.0).[24] The surrogate variable analysis method allows for the estimation of variation due to hidden or unmeasured factors while also explicitly accounting for known variables. This ensures that variation accounted for by the surrogate variables is distinct from the known covariates such as sex. Residual expression values after regressing out all surrogate variables and sex were used for downstream analyses. Cis-eQTLs analysis was performed using QTLtools (Version 1.1) with a *cis*-distance limit of 1,000,000 base pair (1 Mb) from each gene.[25] The top eQTL SNP per gene was defined as the SNP with the lowest p value for that gene.

GWAS Summary Data

GWAS summary data for 46 metabolites were downloaded from the Metabolomics GWAS Server.[20,26] Metabolites for this analysis were selected based on having GWAS-significant loci where at least one gene was identified as having a plausible or established biochemical link to the associated metabolite. Unknown metabolites and metabolite ratios were excluded from this analysis.

LASSO Regression, Model Filtering, and Final Model Selection

LASSO regression—a multivariate penalized regression procedure that simultaneously performs feature selection along with model fitting[27]—was used to select an optimal set of SNPs for explaining the expression of each gene. Regression was performed using the R package glmnet on each gene, with all SNPs less than 1 Mb from the gene's transcription start or end site as potential covariates.[27] To select the optimal penalty factor for each gene, mean squared error (MSE) was calculated using 10-fold cross-validation across 100 automatically selected potential penalty factors. Given that data partitioning is random for cross-validation, this process was repeated 200 times per gene, and the penalty factor that had the mean lowest MSE across all iterations was selected as recommended in the reference manual for glmnet.

For genes with multiple SNPs selected by LASSO regression, all selected SNPs were first linearly modeled against the gene's expression. Model R^2 was calculated for the full linear model. Iteratively, starting with the SNP with the lowest p value in the model, SNPs were added back one at a time, each time calculating the subset model's R^2 (i.e., forward regression). For groups of SNPs in perfect LD, one was randomly selected and retained. Once 95% of the full

model's R^2 value was attained, any SNPs not in the current subset model were eliminated. The final subset of SNPs was then modeled against expression and smoothed using ridge regression to minimize overfitting, and penalty factors were selected using 25 iterations of 10-fold cross-validated ridge regression. For genes with only one SNP selected by LASSO, this SNP alone was modeled against gene expression using 25 iterations of 10-fold cross-validated ridge regression. The final coefficients from ridge regression models were carried forward for use in S-PrediXcan. Model fit p values were determined by modeling pre-validated predicted expression of each gene against the observed expression. Model fit p values were false-discovery-rate- (FDR)-corrected study wide (all genes and all tissues simultaneously), and those with adjusted p values ≥ 0.01 were excluded from further analysis due to poor model fit.

TWAS with S-PrediXcan

For each modeled gene, Summary-PrediXcan (S-PrediXcan, version 0.5.4) was used to calculate a Z score, which is a linear model of SNP effects for all SNPs in the gene's final ridge regression model described above.[14] The S-PrediXcan method is an extension of PrediXcan that allows the use of summary statistics from GWAS. Each SNP's effect is the product of its expression association coefficient from the prediction model, its GWAS Z score from the summary statistics, and a SNP variance term (the SNP's standard error divided by the standard error of the gene's predicted expression). The SNP expression association coefficients used were those resulting from the final filtered gene expression ridge regression models. GWAS Z scores were calculated manually from effect size and standard error because some SNPs had published summary statistics with GWAS p values of 0 due to rounding. The S-PrediXcan formula was implemented as follows:

$$Z_g \approx \sum_{l \in Model_g} w_{lg} \frac{\widehat{\sigma}_l}{\widehat{\sigma_g}} \frac{\widehat{\beta}_l}{se\left(\widehat{\beta}_l\right)}$$

where w_{lg} is the weight of the SNP l in the expression model for gene g, $\widehat{\sigma}_l$ is the variance for SNP l, $\widehat{\sigma_g}$ is the estimated variance of the predicted expression for gene g,$\widehat{\beta}_l$ is the GWAS regression coefficient for the SNP, and $se(\widehat{\beta}_l)$ is the GWAS standard error.[14]

Conditional Analysis

For significant genes identified by S-PrediXcan, we decomposed the Z scores into per-SNP scores. For each significant gene, for SNPs from the S-PrediXcan model that had the same individual direction of effect as the overall S-PrediXcan Z score, the SNP that had the highest absolute S-PrediXcan magnitude was considered the top contributing SNP for conditional analysis. Conditional analysis was performed on each significant S-PrediXcan gene using GCTA (version 1. 26.0).[28] Each lead SNP effect was conditioned out of the GWAS summary data. S-PrediXcan was then performed as previously described, excluding the SNP or SNPs being conditioned on, and using the GWAS Z scores resulting from the conditional GWAS analysis.

Performance Benchmarking and Establishing Biological Plausibility of Novel Genes

A "truth" set of 61 genes with a previously established biological link to the associated metabolite genes[20] was used to estimate the sensitivity and PPV of our TWAS analyses for detecting true positive (TP) gene-metabolite pairs. Although both sensitivity and PPV can be directly estimated from the associated results, estimating specificity is complicated by the fact that the number of true negatives (TNs) required for this calculation is unknown and would require multiple assumptions to estimate. For example, assumptions would need to be made about the relevant criteria for determining the total number of candidate genes at met-QTL regions. These criteria could include the appropriate genomic range, evidence of genes being genetically regulated, or prior evidence of genes being involved in metabolic functions. Moreover, the total number of candidates varies greatly when considering all possible gene-metabolite pairs compared to more modest assumptions (e.g., assuming that each gene within a region may associate with at least one metabolite). Due to the vulnerability of specificity estimates to underlying assumptions relevant to the number of TNs, we therefore focused our TWAS benchmarking on sensitivity and PPV estimates.

To assess the biological plausibility of novel associations from TWAS (i.e., gene-metabolite pairs that were not in the "truth set" curated by Shin et al.[20]), annotated protein information was downloaded from the Human Metabolome Database (version 3.6) on December 11, 2017.[29] HUGO gene names, metabolism pathways, and gene ontology classifications listed in this database were referenced to assess membership of significant S-PrediXcan-associated genes. Metabolic pathways and gene ontology (GO) classifications annotated to novel genes were compared with those for putative causal genes associated with the same metabolites in order to assess shared metabolic processes.

Results

Multi-SNP Models Explain More Variance in Gene Expression than Do Single-eQTL Models

To investigate gene associations based on multi-SNP models, we first evaluated the extent to which these models improve prediction of gene expression relative to single-variant models. We obtained single-variant models by performing standard univariate eQTL analysis to identify the top associated *cis*-SNP for each gene in each of 43 tissues from the GTEx study (version 7) with a sample size exceeding 50 (see Material and Methods).[22] The number of expressed genes (defined as genes with >6 raw reads and >1 count per million in at least 10 individuals) ranged from 15,483 in EBV-transformed lymphocytes to 19,846 in lung tissue.

To obtain multi-SNP genetic prediction models of gene expression, we employed LASSO regression to select an optimal and sparse set of *cis*-SNPs to jointly model expression of each gene in each tissue. We then compared the variation in gene expression explained by these multi-SNP models to that accounted for by the single eQTL models.

In Figure 1, we show representative results, in this case for skeletal muscle, the tissue with the largest sample size (n = 491). LASSO regression selected multiple SNPs in the models for the majority of genes (n = 11,210), and for these genes, there was a median of 2.4-fold increase (interquartile range [IQR], 1.7- to 3.9-fold) in expression variation explained by LASSO models versus the top eQTL alone (Figures 1A and 1B). There was a 2.0-fold median increase in expression variation explained across all gene models (i.e., including single-eQTL models) in

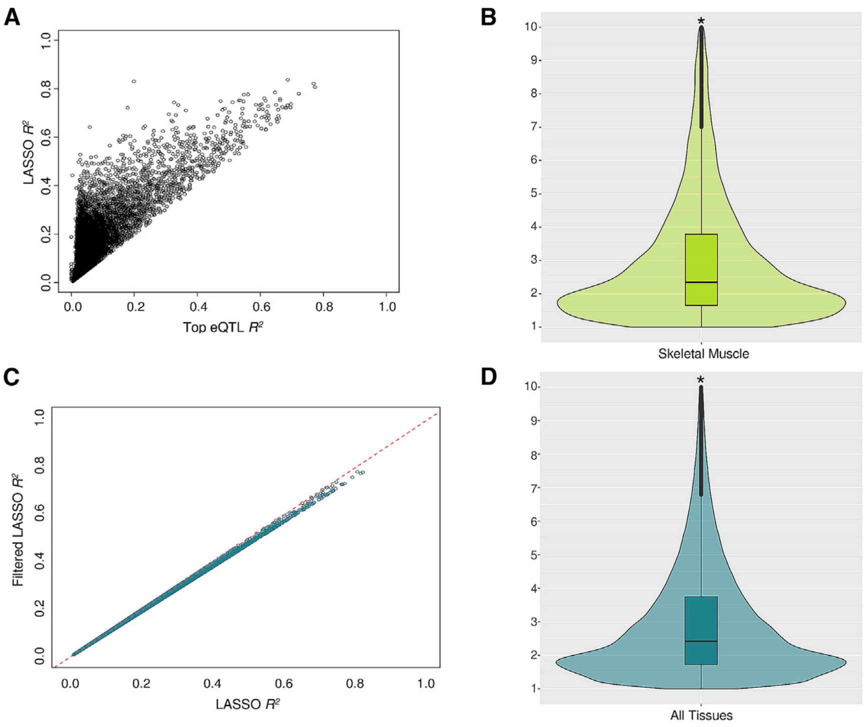

Figure 1. Model R^2 Comparison of LASSO Regression Models
(A) Scatterplot comparing variation in gene expression explained by the top eQTL alone and by the multi-SNP LASSO model in skeletal muscle.
(B) Violin plot showing the fold increase in gene expression variation explained by LASSO models in skeletal muscle. The asterisks in the violin plot denote that the y axis is abrogated at a fold change of 10. The box plot indicates the median and IQR of fold increase in gene expression.
(C) Comparison of LASSO regression models before and after contribution-based filtering of collinear SNPs. The mean model R^2 was reduced by only 1.6%.
(D) Violin plot showing the fold increase in gene expression variation explained by filtered LASSO models across all 43 tissues. The asterisks in the violin plot denote that the y axis is abrogated at a fold change of 10. The box plot indicates the median and IQR of fold increase in gene expression.

skeletal muscle. LASSO selected the intercept-only model (i.e., model without any SNPs) for 2,667 genes out of 15,780 expressed genes, and the top eQTL-only model (or a perfect proxy SNP) for 1,903 genes in skeletal muscle. The impact of multi-SNP selection seen for skeletal muscle was typical of that seen across all tissues and all genes (Table S1).

Despite the sparse nature of LASSO selection, resultant expression models contained up to 159 SNPs and a median of nine SNPs (IQR, four to 18 SNPs) for models with >1 SNP. Moreover, for those genes with at least two modeled variants, 7,406 genes (66%) in skeletal muscle retained at least one pair of SNPs with LD $r^2 > 0.8$. Since correlated SNPs can result in invalid inference for summarized MR analyses,[30] we performed additional filtering of SNPs based on LD and proportion of variation explained (R^2), iteratively adding SNPs into the model until 95% of the full model's R^2 was achieved. At each step of this procedure, for groups of SNPs in perfect LD ($r^2 = 1$), one SNP was randomly selected and retained (see Material and Methods). This reduced the median number of SNPs per gene in the model in skeletal muscle to six (IQR, three to 12 SNPs, Table S1). Moreover, 18% of gene models (2,015 out of 11,210 models that included multiple SNPs in the unfiltered analysis) contained only the top eQTL (or a perfect proxy). In addition, there was an overall reduction in the number of gene models with collinear SNPs: of the 7,406 genes with pairs of SNPs in high LD ($r^2> 0.8$) from LASSO regression, only 306 models contained a pair of SNPs in high LD after the stepwise filtering procedure. This further round of filtering had little impact on model performance; the mean reduction in model R^2 was only 1.6% (calculated as percentages of the full LASSO models' R^2 values; Figures 1C and 1D). Similar to the results for unfiltered LASSO models in skeletal muscle, there was still a 2.0-fold median increase in expression variation explained across all gene models and across all tissues. Overall, these results demonstrate that multi-SNP models—even after optimization to reduce model complexity and minimize collinearity—explained substantially more of the variation in gene expression than did the equivalent single-SNP models across tissues.

Although the majority of individuals in GTEx v7 were self-identified as "White," 14% were of non-European (or unknown) ancestry, and the majority of those (12%) were African American. It has been shown that gene expression prediction models trained on data from one ancestry group perform less well when used to impute expression in other ancestry groups.[31,32] To assess whether differences in LD between major ancestral groups had an effect on model fitting, we generated filtered LASSO gene expression prediction models for skeletal muscle from the subset of individuals recorded as "White" in GTEx v7 (421/491 individuals) and compared these to filtered models generated from all individuals. Results obtained in the "White" subset were almost identical to those in the full dataset. We found that (A) the proportion of gene expression variation explained was highly correlated (Pearson correlation of 0.88); and (B) there was a similar fold-increase in the expression variation explained by multi-SNP models compared to the single best eQTL, corresponding to a median 2.3-fold increase (IQR of 1.5- to 4.0-fold) in "White" individuals that was consistent with prediction models from all individuals (median of 2-fold increase, IQR of 1.3- to 3.5-fold). Because these results

indicated that ancestral differences in LD properties among model SNPs were modest, and in the interest of maximizing predictive power, we carried the LASSO models trained from the full GTEx dataset forward for downstream analyses.

TWAS of 46 Metabolites across 43 Tissues

Given these estimates of the extent to which multi-SNP models enhance the prediction of gene expression, we next sought to assess their utility in understanding genetic variation associated with complex diseases and traits. Metabolites offer a singular opportunity for such analyses because recent GWAS have identified strongly associated loci that regulate metabolite levels (met-QTLs).[18–21]

At some of these loci, extensive genetic and experimental evidence has identified nearby genes for which the biological evidence for a causal role in mediating the metabolomics association is overwhelming, providing a "truth" set for causal gene localization not available in most other trait GWAS settings.

We focused on 46 metabolites with publicly available GWAS summary data for which at least one gene mapped near a significant met-QTL signal with high-confidence biochemical links to the associated metabolite (Table S2).[20] Because individual-level genotype and phenotype data are not easily accessible for most large-scale GWAS studies, methods that estimate TWAS associations using summary GWAS data and genetic reference panels have been widely adopted.[5,13,14] We therefore performed TWAS with S-PrediXcan[14]—an extension of PrediXcan that allows the use of summary statistics from GWAS—to test for associations between predicted gene expressions across 43 tissues and these 46 metabolite levels. Analysis was restricted to filtered LASSO prediction models with a strict significant expression model fit (model $q < 0.01$; $n = 568{,}185$ total gene models).

A total of 2,834 associations between predicted gene expression values and metabolite levels reached significance at study-wide FDR < 0.01, corresponding to 826 unique gene-metabolite pairs (i.e., some pairs were significantly associated in multiple tissues) (Figure 2A). The largest number of associations identified for any tissue was 100 (tibial nerve). There were only 66 associations arising from predictive models generated from liver expression data (8% of 826 unique associations), even though liver could be considered the most biologically relevant tissue for most of these metabolites. This is likely due to the relatively small sample size for liver in GTEx (153 samples compared to 361 in tibial nerve) (Figure 2B, Table S1, Table S3).

For these 826 unique gene-metabolite pairs, we next sought to understand the extent to which multiple independent SNPs selected by the model were contributing to these metabolite associations. To do this, we performed conditional analyses for each of the 2,593 (from the total of 2,834) significant S-PrediXcan associations where the gene model had more than one SNP. We conditioned the metabolite GWAS on the SNP with the greatest effect on each gene's S-PrediXcan score, and we re-ran the S-PrediXcan association test using the conditioned GWAS summary statistics. After we corrected for the number of genes, tissues, and metabolites tested after conditional analysis (p value$_{\text{conditional}} \leq 1.93\text{x}10^{-5}$), 2,320 of the 2,593 associations (89.5%) were no longer significant. This proportion was similar if we instead analyzed only the most significant tissue for each gene; 684 out of 758 gene-metabolite pairs (90.2%) were no longer significant (p value$_{\text{conditional}} \leq 6.61\text{x}10^{-5}$). Thus, for over 90% of significant S-PrediXcan associations, evidence for mediation of metabolite levels was dominated by a single SNP within the multi-SNP prediction models. Of the 273 (of 2,593) signals that were still significant after conditioning on the lead SNP, over half (148) involved genes within 1 Mb of the highly complex *ACADS* gene region, which features multiple independent met-QTLs significantly associated with butyrylcarnitine levels (Figure 3, Table S4).

Colocalization Analysis of Model SNPs Reveals the Distinct Relationships between *cis*-eQTL and met-QTL Signals

It is possible that overlaps between GWAS met-QTLs and *cis*-eQTL variants in multi-SNP models could be due to chance, rather than representing true colocalization of causal signals. Consider, for example, a multi-SNP model with two SNPs where one SNP is a strong eQTL but weakly associated with metabolite levels, and the other SNP displays the converse arrangement: this configuration could still yield a significant association between gene expression and metabolite levels. We therefore questioned to what extent multi-SNP S-PrediXcan associations were driven by *cis*-eQTL and met-QTL signals that shared the same identity (i.e., the associations were attributable to SNPs that influence metabolite levels through their effects on gene expression).

We addressed this by performing colocalization analysis using eCAVIAR to obtain colocalization posterior probability (CLPP) values as evidence of shared causal signals, benefiting from the fact that eCAVIAR allows for multiple causal variants within a locus.[8] To increase our power to detect genuine colocalization, we restricted this analysis to those SNPs in the prediction models that were significant *cis*-eQTLs (per tissue FDR < 0.01) and met-QTLs (GWAS p value $\leq 5.0\text{x}10^{-8}$).

We found that, among the 2,834 significant S-PrediXcan associations, about 35% of associations (990; 214 unique gene-metabolite pairs) contained at least one SNP in the prediction model that influenced both metabolite levels at genome-wide significance and expression levels at FDR < 0.05. Of these, 907 associations (92% of 990 associations tested; 202 unique gene-metabolite pairs) had at least one significant *cis*-eQTL with a CLPP > 0.01, evidence of a shared causal signal between met-QTL and *cis*-eQTL, in at least one tissue[8] (Table S5). Therefore, for the SNPs that corresponded to gene models and that were amenable to colocalization analysis, there was strong evidence of shared eQTL and met-QTL signals.

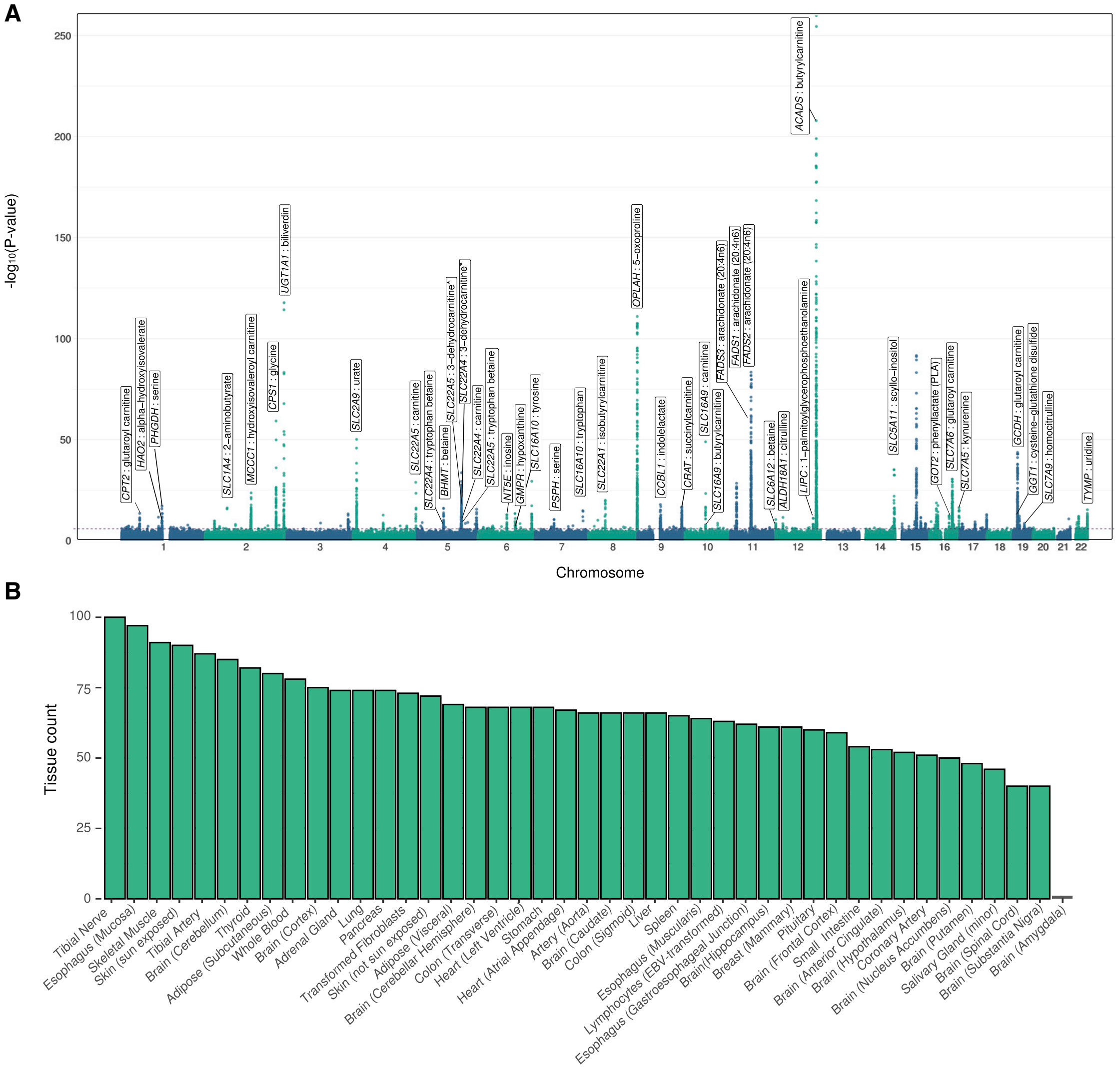

Figure 2. Transcriptome-wide Association Studies of 46 Metabolites across 43 Tissues
(A) Manhattan plot showing all S-PrediXcan associations across 46 metabolites in all 43 tissues analyzed, with each point representing a gene-metabolite association. Labels indicate loci where TWAS associations involve high-confidence causal genes. The y axis shows the negative log10 p values from the S-PrediXcan association test.
(B) Bar plot of the number of significant gene-metabolite associations observed per tissue.

We then analyzed the context within which *cis*-eQTL SNPs in the multi-SNP models colocalized with met-QTLs. For the 907 associations with evidence of colocalization, we observed instances of a one-to-one overlap whereby the significant *cis*-eQTL in the multi-SNP model colocalized with the corresponding met-QTL. An example of this arrangement is displayed in Figure 4A. However, determining the evidence for or against colocalization of the met-QTL and *cis*-eQTLs was not always as simple, because many loci had a more complex topography. For example, expression of *SLC16A9* was significantly associated with carnitine levels in S-PrediXcan analyses in tibial nerve. Two significant *cis*-eQTLs with low LD ($r^2 = 0.002$) were selected in the prediction model, but, as the locus plot shows, only one of these signals colocalized with the met-QTL (Figure 4B).

In contrast, we observed significant TWAS associations where model SNPs had divergent effects on expression and metabolite levels and were thereby excluded from colocalization analysis (i.e., associations not included in the 907 associations with evidence of colocalized QTL signals). For example, the expression of *FNDC1* in skeletal muscle was significantly associated with circulating isobutyrylcarnitine levels. However, the met-QTL and *cis*-eQTL were clearly not colocalized even though the genetically predicted expression of *FNDC1* was significantly associated with metabolite levels. This is because the set of SNPs in the *FNDC1* prediction model includes both the SNP driving the strong met-QTL (which explains a small portion of the variance in *FNDC1* expression) and a strong *cis*-eQTL that is only weakly associated with metabolite levels (Figure 4C).

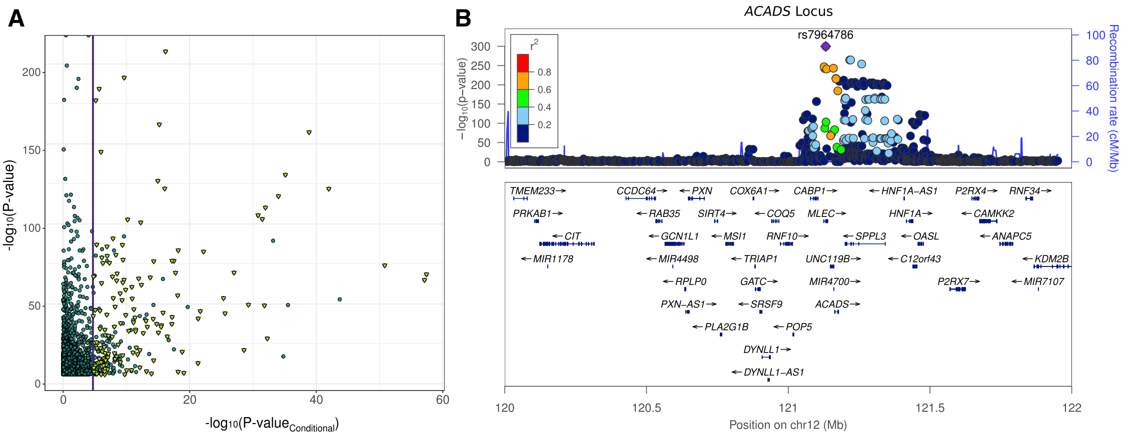

Figure 3. Stepwise Conditional Analysis of Significant Associations
(A) Plot showing results from the conditional analysis of S-PrediXcan associations involving multi-SNP prediction models. The vertical line denotes the significance threshold used for conditional analysis. Only 273 associations remained significant after conditioning on the lead met-QTL SNP, of which 148 mapped to the *ACADS* locus and influence butyrylcarnitine levels (yellow triangles). The x and y axes correspond to negative log10 p values from the conditioned and unconditioned S-PrediXcan association test.
(B) Locus zoom plot showing met-QTLs associating with butyrylcarnitine levels at the *ACADS* locus and their LD relative to the top met-QTL.

Determining the Sensitivity and PPV of Multi-SNP Prediction Models

Across the GWAS of 46 metabolites that we used as the substrate for our analyses, Shin et al. previously reported 61 SNP-metabolite associations at which the associated met-QTL SNP mapped near a gene that was highly likely to be causal for the association. This assessment was based on either experimental validation or a strong biological plausibility that the encoded protein was involved in the synthesis or degradation of the metabolite concerned.[20] These 61 SNP-gene-metabolite groupings provide a "truth" set of causal genes that can be used to explore the performance of expression-QTL-based mapping strategies, information relevant to more common applications (e.g., in a disease GWAS) where the causal gene is typically not known with equivalent certainty.

Of these 61 gene-metabolite pairs in the "truth" set, we were able to detect 41 through significant S-PrediXcan associations in at least one GTEx tissue (Table 1); this result indicates a sensitivity for *cis*-eQTL validation of the causal gene of 67%. Thirty-three of these gene-metabolite assignments were supported in more than one tissue, and the *GCDH*-glutarylcarnitine association was the most widely represented (detected in 38 tissues, Table 1). Only eight of the 41 were detected in liver, though this may in part reflect the relatively small sample size of liver in GTEx (Figure 2B, Table S1, Table S3). We assessed the extent to which overlaps between eQTLs and GWAS at these truth set genes represented true colocalization of signals. Of these 41 genes, 23 were amenable to colocalization analysis (i.e., at least one of the SNPs in the model was a significant *cis*-eQTL and a significant met-QTL) and all of these 23 genes showed evidence of colocalization, where at least one SNP in the multi-SNP model colocalized with the met-QTL in at least one tissue.

As described earlier, our genome-wide trawl for associations between metabolite levels and predicted expression levels across GTEx tissues had implicated 826 unique gene-metabolite pairs. Of these, more than half (514; 62%) involved genes that mapped within 1 Mb of the 61 truth set genes (including the 41 detected truth set genes). This indicates that, at many of these loci, there are multiple "bystander" genes, other than the truth set genes, which are also being detected through predicted expression. At only four of the truth set loci did these analyses identify the true causal gene only with no such bystander genes.

From this analysis of TWAS associations at metabolite-associated loci, we estimate that the PPV (i.e., the number of TPs divided by the sum of true and false positives) for detecting TP associations is only 8% (41/514 gene-metabolite pairs). (We focus on PPV rather than specificity because estimates of specificity are heavily dependent on assumptions regarding the set of TNs, as described in Material and Methods.) One possible explanation for this low PPV is LD-tagging (where the metabolite associated variants are distinct from the cis-eQTLs but are correlated through LD), and it has been suggested that tests of colocalization can be used to separate out spurious from consequential gene assignments from TWAS.[33,34] To test this, we repeated these analyses, limited to the 214 gene metabolite associations amenable to colocalization analysis (using the same parameters described above). Of these, 23 involved "true" causal genes, all of them colocalized, and 105 involved bystander genes in the same regions (95 colocalized); the result, in this subset, was an improved PPV of 19% (23/(23+95)). This modest improvement in PPV came at the price of a substantial reduction in sensitivity, from 67% to 38% (23/61 true causal genes detected with evidence of colocalization).

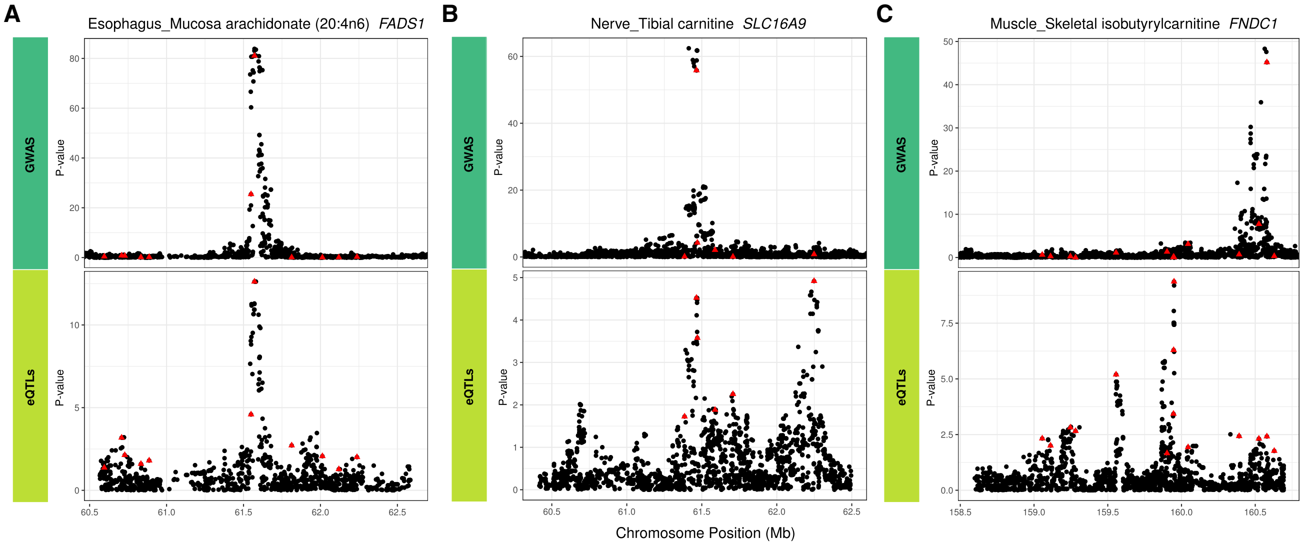

Figure 4. Colocalization Analysis of eQTL and met-QTL Signals in Multi-SNP Models for Metabolite-Associated Genes
(A) Colocalization of the single met-QTL and single *cis*-eQTL signal at the *FADS1* gene in esophagus mucosa.
(B) Partial colocalization at the *SLC16A9* gene in tibial nerve where only one of the two independent *cis*-eQTLs in the multi-SNP model is colocalized with the met-QTL at this gene.
(C) No colocalization of *cis*-eQTL and met-QTL for the *FNDC1* gene in skeletal muscle. The red triangles denote the SNPs present in the genes' multi-SNP prediction models.

20 of the 61 gene-metabolite pairs in the truth set did not yield significant S-PrediXcan associations in any tissue. However, for 15 of these, significant S-PrediXcan associations (from the set of 514 gene-metabolite pairs described above) were seen for nearby bystander genes in at least one tissue, with eight of these showing significant bystander gene colocalization (Table S5). Taken together with the results for the 41 TP signals, these analyses indicate substantial pleiotropy at the level of *cis*-eQTLs, with many met-QTL loci harboring a substantial excess of "bystander" genes alongside the true causal gene (or at some loci, only "bystander" genes).

To illustrate these concepts, consider SNP rs8012, which is a significant met-QTL for glutarylcarnitine levels (p value$_{GWAS}$ = 1.24x10^{-43}), and maps 8 kb from *GCDH* that encodes the enzyme glutaryl-CoA dehydrogenase. This enzyme catalyzes the conversion of glutaryl-CoA to crotonyl-CoA, making *GCDH* a highly plausible effector gene mediating the effects of rs8012 on glutarylcarnitine levels.[35] In GTEx, while rs8012 is a *cis*-eQTL for *GCDH* in 31 tissues, the same SNP is also associated with the expression of other nearby genes including *HOOK2, SYCE2, FARSA, AD000092.3,* and *CALR*. For all these genes, the *cis*-eQTL and the met-QTL signal clearly colocalized in at least one tissue (Figure S1). In the absence of the strong biological prior favoring *GCDH* at this locus, at least five other genes could be equally plausible candidates.

We next asked whether there were any features of the 473 bystander genes that might allow them to be distinguished from truth set genes. We found that bystander genes did not differ with respect to the strength of association with the metabolite, the distance to the transcription start site, the effect sizes of the individual eQTLs included in the multi-SNP models, or the CLPP values for model SNPs (Figure 5). However, we did find that causal genes tended to be significant in more tissues than did bystander genes at the same locus (Figure S2).

In addition to the 61 SNP-metabolite pairs in the truth set, Shin et al. reported 18 SNP-metabolite pairs that reached genome-wide significance in their analysis, but for which it was not possible to assign a causal gene with high confidence, because none of the genes could be implicated based on known biology.[20] In this setting, the authors assigned each associated SNP to the nearest gene at the locus (Table S2). The results of our analyses for these 18 signals recapitulated those we saw for the 61 genes in the "truth set." We could recover 10 of these "nearest gene" candidates (a sensitivity of 56%), of which seven colocalized in at least one tissue, through the use of S-PrediXcan applied to GTEx (Table S6). However, a further 92 bystander associations at these loci were also significant.

We also used a complementary approach to quantify the performance of the predicted expression analysis for identifying novel genes (i.e., genes corresponding to gene-metabolite pairs that were not in the "truth set" curated by Shin et al.) that are biologically plausible. We focused on the 312 gene-metabolite pairs that involved genes that did not map to known met-QTL regions, and we evaluated metabolite and gene annotations in the Human Metabolome Database (version 4.0).[29] We found that 96 of these pairs—corresponding to 83 genes—involved genes annotated to metabolic pathways. These included two genes involved in uridine metabolism: *CDA* and *UPP1*. Notably, SNPs at these two loci were sub-genome-wide

Table 1. **Causal Genes from the Truth Set That Significantly Associated with Metabolite Levels in a TWAS**

Metabolite ID	Metabolite Name	Causal Gene	Number of Associations	Most Significant Tissue	q-Value
M35439	glutarylcarnitine	*GCDH*	38	whole blood	1.88×10^{-39}
M01110	arachidonate (20:4n6)	*FADS1*[a]	27	thyroid	1.09×10^{-78}
M32412	butyrylcarnitine	*ACADS*[a]	26	lung	5.64×10^{-202}
M01110	arachidonate (20:4n6)	*FADS2*	26	esophagus gastresophageal junction	1.76×10^{-48}
M37058	succinylcarnitine	*CRAT*[a]	23	cells transformed lymphocytes	5.78×10^{-13}
M00606	uridine	*TYMP*	20	cells transformed fibroblasts	1.36×10^{-11}
M35433	hydroxyisovaleroyl carnitine	*MCCC1*	16	skin sun exposed	1.29×10^{-9}
M01604	urate	*SLC2A9*	14	muscle skeletal	3.57×10^{-34}
M03141	betaine	*BHMT*	11	brain frontal cortex	3.21×10^{-12}
M32338	glycine	*CPS1*	10	brain putamen	3.15×10^{-10}
M32654	3-dehydrocarnitine	*SLC22A5*[a]	10	skin not sun exposed	5.64×10^{-14}
M01123	inosine	*NT5E*[a]	8	spleen	3.19×10^{-9}
M15500	carnitine	*SLC16A9*	8	esophagus mucosa	1.28×10^{-44}
M15140	kynurenine	*SLC7A5*	8	adipose visceral	1.58×10^{-12}
M22138	homocitrulline	*SLC7A9*	7	colon transverse	0.000202
M01110	arachidonate (20:4n6)	*FADS3*[a]	6	liver	9.96×10^{-55}
M35159	cysteine-glutathione disulfide	*GGT1*	6	esophagus mucosa	1.88×10^{-8}
M35439	glutarylcarnitine	*SLC7A6*	6	spleen	4.24×10^{-14}
M35439	glutarylcarnitine	*CPT2*	5	colon sigmoid	8.48×10^{-8}
M01494	5-oxoproline	*OPLAH*	4	skin sun exposed	5.08×10^{-98}
M02137	biliverdin	*UGT1A1*[a]	4	skin not sun exposed	1.16×10^{-49}
M32315	serine	*PHGDH*	3	colon sigmoid	2.73×10^{-13}
M33441	isobutyrylcarnitine	*SLC22A1-2*	3	skin not sun exposed	3.20×10^{-5}
M32654	3-dehydrocarnitine	*SLC22A4*	3	skin sun exposed	1.06×10^{-17}
M15500	carnitine	*SLC22A4*	3	artery tibial	2.01×10^{-7}
M15500	carnitine	*SLC22A5*	3	brain cerebellum	0.00104
M37097	tryptophan betaine	*SLC22A5*	3	brain putamen	2.35×10^{-5}
M18349	indolelactate	*CCBL1*	2	brain cortex	0.000201
M03127	hypoxanthine	*GMPR*	2	brain cerebellar hemisphere	0.00228
M22130	phenyllactate (PLA)	*GOT2*	2	brain frontal cortex	1.05×10^{-8}
M35631	1-palmitoylglycerophosphoethanolamine	*LIPC*[a]	2	pancreas	3.96×10^{-6}
M03141	betaine	*SLC6A12*	2	lung	0.00148
M02132	citrulline	*ALDH18A1*	1	skin sun exposed	0.00807
M33937	alpha-hydroxyisovalerate	*HAO2*	1	adrenal gland	1.53×10^{-6}
M32315	serine	*PSPH*	1	esophagus mucosa	0.000534
M00054	tryptophan	*SLC16A10*	1	brain frontal cortex	0.00671
M01299	tyrosine	*SLC16A10*	1	brain frontal cortex	0.00058
M32412	butyrylcarnitine	*SLC16A9*	1	esophagus mucosa	0.000185

(*Continued on next page*)

Table 1. *Continued*

Metabolite ID	Metabolite Name	Causal Gene	Number of Associations	Most Significant Tissue	q-Value
M32348	2-aminobutyrate	*SLC1A4*	1	muscle skeletal	1.96×10^{-12}
M37097	tryptophan betaine	*SLC22A4*	1	artery tibial	3.28×10^{-7}
M32379	scyllo-inositol	*SLC5A11*	1	brain hippocampus	0.00474

Of the 61 high-confidence truth set genes, 41 had significant S-PrediXcan associations in at least one tissue.
[a]Eight gene-metabolite pairs that had a significant association in liver.

significant in the GWAS but were implicated from our S-PrediXcan analysis and subsequent studies[17] (Table S7). We expanded the search further by querying a recently curated dataset,[17] and we found an additional 18 genes annotated to at least one metabolic pathway. Thus, as many as 37% (114/312 gene-metabolite pairs) of novel TWAS gene associations can be considered biologically plausible, albeit based on the rather permissive overlap between "metabolic pathway" and met-QTL.

We then performed a more stringent evaluation by determining the number of novel gene-metabolite associations (again excluding "bystander" genes) where the novel gene either shared at least one metabolic pathway with a reported truth set gene for the associated metabolite or has been curated as a high-confidence causal gene with the associated metabolite in recent publications.[17] We found that 16 (5%) of the 312 novel gene-metabolite pairs met this criterion (Table S8). Taking this as a lower limit and the previous, less stringent estimate as an upper limit, we estimate that 5%–37% of novel gene-metabolite relationships are biologically plausible. Notably, this range encompasses our PPV estimate of 8%, which was obtained by evaluating the TP rate at met-QTLs with known causal genes. Therefore, most novel gene associations based on multi-SNP models represented either false positives or "bystander" genes that are not biologically relevant per se but rather are driven by variants with pleiotropic effects on gene expression. Overall, these findings emphasize that, while the multi-SNP *cis*-eQTL approach has respectable sensitivity in detecting the causal gene in these data, performance in terms of PPV is poor and additional lines of evidence will be needed at most loci to establish causality.

Discussion

In this study, we have assessed the utility of multi-SNP prediction models for explaining variation in gene expression and the application of these models in TWAS. We quantified the extent to which these models outperform expression models based on a single eQTL, demonstrating, across all evaluated tissues, a median 2-fold improvement in variance explained. When applied in a TWAS of genome-wide data for 46 metabolites across 43 human tissues, these multi-SNP models identified 826 significant gene-metabolite associations. By leveraging knowledge of genes highly likely to be causally involved in the regulation of metabolite levels, we were able to quantify the accuracy with which multi-SNP TWAS detects such high-confidence effectors. The results from these analyses offer several key insights relevant to the interpretation of TWAS results.

We found that, although the use of LASSO regression is a sparse form of variable selection, it still tends to select sets of SNPs that are highly correlated, introducing multicollinearity into resulting regression models. This notion has been described before in real and simulated GWAS data.[36] We showed that a simple iterative approach to LASSO modeling that involved LD-based filtering resulted in increased model sparsity and decreased multicollinearity, leading to more confident genetic instruments for gene expression.

Despite the improved performance in predicting gene expression attributable to models with multiple, independent SNPs, we found that, using available GTEx data, TWAS associations based on these models were, in most instances, driven by a single SNP within each trait-associated locus: 90% of associations were no longer significant after stepwise conditional analysis. This is consistent with previous studies that showed that lead eQTLs explained a disproportionately high share of the heritability of gene expression in peripheral blood and that top eQTLs also explain a large proportion of heritability for multiple complex traits.[37,38] Although this proportion is likely to fall as eQTL sample sizes increase (increasing the power to detect the additional impact of conditioned variants), these results indicate that, for many genes, the increment in power gained by moving from single- to multi-SNP analyses is modest.

The genetic architecture underlying metabolite traits provides a unique opportunity to quantify the performance of gene associations based on multi-SNP models. By leveraging a "truth" set of experimentally validated genes linked to metabolites,[20] we have shown, using GTEx, that TWAS has reasonable sensitivity (67%) for identifying causal genes. However, the PPV is low (8% rising to 19% if combined with evidence of colocalization); a great majority of associations in the vicinity of a known causal gene involved nearby "bystander" genes.

Furthermore, the process of resolving true causal from false-positive associations is complicated by the fact that these types of associations were indistinguishable in their model SNP effect sizes (GWAS and eQTL), colocalization

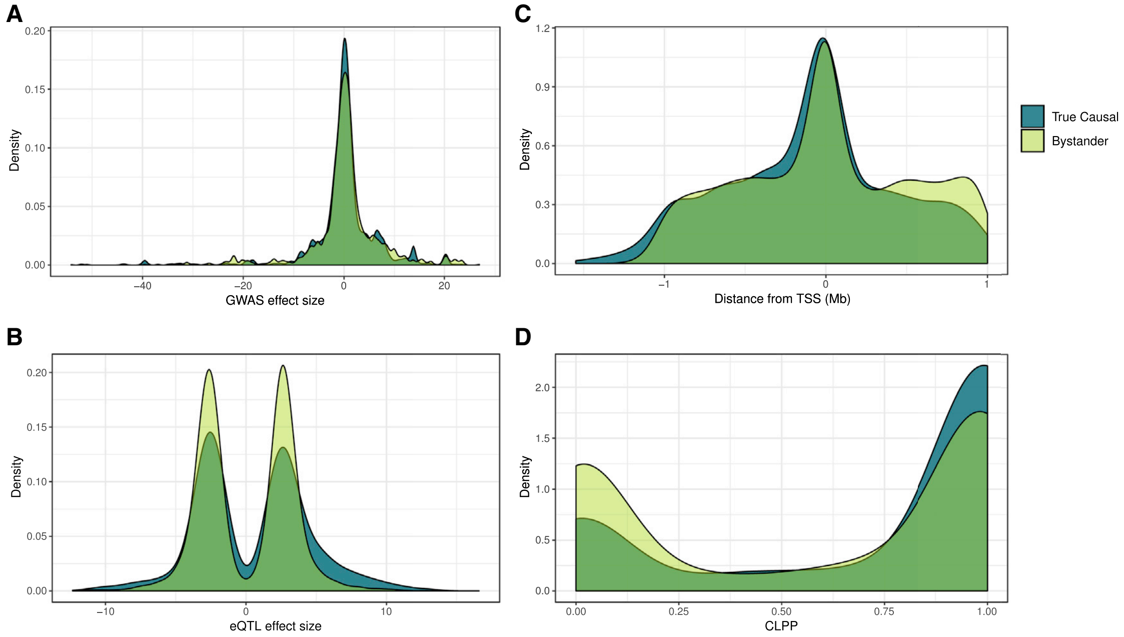

Figure 5. Comparison of Features of Multi-SNP Models for Bystander Genes to Those for True Causal Genes
(A) Comparison of the effect sizes of model SNPs for bystander genes and model SNPs for true causal genes on metabolite levels in GWAS.
(B) Distribution of effects on gene expression for individual SNPs in models for bystander and known causal genes.
(C) Comparison of the distance from TSS for model SNPs in bystander and causal genes.
(D) The distribution of colocalization posterior probability (CLPP) scores for model SNPs in bystander and causal genes.

probabilities, and distance to transcription start sites. In the case of the metabolite glutarylcarnitine, for example, the met-QTL rs8012 regulates not only the expression of the causal *GCDH* gene but also the expressions of five other genes at the same locus, all of which are associated with glutarylcarnitine levels in TWAS. These insights temper the extent to which it can be assumed that genes implicated by significant TWAS associations are causal.

These "bystander" effects reflect their shared regulatory architecture with known causal genes, and our observations around met-QTLs mirror recent findings at the *SORT1* and *NOD2* loci (associated with LDL cholesterol and Crohn's disease, respectively).[39] By anchoring our analysis on a wide range of metabolomic phenotypes, we have been able to extend those observations and to develop more generalizable estimates of the sensitivity and PPV of TWAS. Recent analyses from Stacey and colleagues using an alternative gene prioritization method (ProGeM) are also instructive.[17] Using ProGeM to address a similar problem (the detection of causal effector genes at met-QTL loci), the performance was appreciably better than that we observed; their results had with a sensitivity of 98% and a specificity ranging from 38.4% to 84.6% (PPV was not measured, and the TNs needed for estimation of specificity were derived using different criteria for delineating sets of candidate causal genes). However, in contrast to TWAS, ProGeM explicitly integrates SNP-level annotations (i.e., eQTLs) with functional gene and pathway annotations across five databases to prioritize causal genes. That is to say, ProGeM directly leverages molecular pathway annotations, whereas TWAS is agnostic to this information. Accordingly, ProGeM is intended for a specific trait class—molecular QTLs (e.g., metabolites, lipids, proteins)—and the incorporation of additional information relevant to metabolites is likely to have contributed to the better performance in this specific task. In addition, the sensitivity of ProGeM may be inflated by the fact that shared database features were used both to prioritize genes and to benchmark performance. For these reasons, ProGeM might be expected not to achieve comparable performance when used to prioritize effectors at disease GWAS loci, with performance more resembling that of the more agnostic approach we achieve with TWAS.

We recognize some limitations of the present study. First, we used the S-PrediXcan approach for TWAS; it is possible that methods such as PrediXcan that use individual-level data could yield fewer false positives, especially where there are mismatches between the GWAS and LD reference populations.[14] Second, we applied a strict stringency threshold (FDR < 0.01) in our TWAS analyses; a more lenient threshold would likely increase sensitivity, albeit with lower precision. Third, while liver is a highly relevant tissue for many of the circulating metabolites in this study, the available sample size for this tissue in GTEx v7 was small, and we correspondingly found relatively few TWAS associations in liver. However, due to the widespread sharing of cis-eQTLs, TWAS analyses that leverage regulatory information across multiple tissues (that may not be immediately relevant to evaluated traits) are able to implicate putative causal genes that are mediating effects in more relevant, yet under-sampled, tissues.[34]

Our analyses were focused on the use of expression QTLs to map causal genes at metabolomic-QTL signals; the extent to which similar observations apply to other molecular QTLs remains to be determined. Previous studies have shown that the genetic architecture of protein-QTLs (pQTLs) is distinct from that of eQTLs; only half of pQTLs identified in lymphoblastoid cell lines (LCLs) were also eQTLs, and pQTL effect sizes were typically lower than those for eQTLs.[40] However, these apparently distinct architectures are likely in part the consequence of disparities in sample sizes and differences in the technologies used to profile these features. Further work is required to assess whether the confounding effect of co-regulation observed in TWAS based on predicted gene expression will be present to the same extent for other molecular features.

TWAS approaches provide an attractive option for prioritizing candidate genes at trait-associated loci. Here, we have demonstrated the potential for these approaches to identify associations that are not causal, through a combination of incomplete colocalization and pleiotropy in gene expression regulation. Ultimately, the process of identifying causal genes at GWAS signals represents an integrative enterprise that is dependent on combining results from multiple complementary approaches, including, in addition to QTL-mapping, epigenome profiling (e.g., chromatin co-accessibility or conformation capture methods), functional screens (e.g., high-throughput gene knockout CRISPR screens), and the detection of coding variant associations. All of these prioritization approaches—including TWAS—will become more accurate as the datasets available encompass a wider range of tissues and cell types captured in circumstances (e.g., developmental stages, physiological states, andenvironmental exposures) that better reflect the underlying pathophysiology of the particular traits and diseases under investigation.

Supplemental Data

Supplemental Data can be found online at https://doi.org/10.1016/j.ajhg.2020.01.003.

Acknowledgments

MMcC is a Wellcome Investigator and a National Institute for Health Research (NIHR) Senior Investigator. Relevant funding support for this work comes from Wellcome (090532, 106130, 098381, 203141, and 212259), the National Institute of Diabetes and Digestive and Kidney Diseases (NIDDK) (U01-DK105535 and U01-DK085545), and the NIHR (NF-SI-0617-10090). A.P. was supported by the Rhodes Trust, the Natural Sciences and Engineering Research Council of Canada, and the Canadian Centennial Scholarship Fund. While employed at the University of Oxford, M.vdB. was supported by a Novo Nordisk postdoctoral fellowship run in partnership with the University of Oxford. This work was also supported by Oxford Biomedical Research Computing (BMRC) facility, a joint development between the Wellcome Centre for Human Genetics and the Big Data Institute supported by Health Data Research UK and the NIHR Oxford Biomedical Research Centre. The views expressed are those of the author and not necessarily those of the National Health Service (NHS), the NIHR, or the Department of Health. We thank Tuuli Lappalainen, Hae Kyung Im, Bogdan Pasaniuc, Eric Fauman, and other colleagues who contributed comments, via social media, to the preprint release of an earlier version of this manuscript that informed subsequent revisions.

Declaration of Interests

M.McC. has served on advisory panels for Pfizer, NovoNordisk, and Zoe Global; has received honoraria from Merck, Pfizer, NovoNordisk, and Eli Lilly; has stock options in Zoe Global; and has received research funding from Abbvie, AstraZeneca, Boehringer Ingelheim, Eli Lilly, Janssen, Merck, NovoNordisk, Pfizer, Roche, Sanofi Aventis, and Servier and Takeda. As of June 2019, M.McC. is an employee of Genentech and holds stock in Roche. M.vdB. has been a full-time employee of Novo Nordisk A/S since May 2017, and he holds stock in Novo Nordisk.

Received: September 20, 2019
Accepted: January 6, 2020
Published: January 23, 2020

Web Resources

Genotype-Tissue Expression (GTEx) Project Portal, https://www.gtexportal.org

Human Metabolome Database, http://www.hmdb.ca/

Metabolomics GWAS Server, http://www. http://metabolomics.helmholtz-muenchen.de/gwas/

The pre-trained multi-SNP models across 43 GTEx (version 7) tissues are available on the McCarthy lab website and on FigShare, http://mccarthy.well.ox.ac.uk/pub/ and https://doi.org/10.6084/m9.figshare.10324055.v1

References

1. Visscher, P.M., Brown, M.A., McCarthy, M.I., and Yang, J. (2012). Five years of GWAS discovery. Am. J. Hum. Genet. *90*, 7–24.
2. Nicolae, D.L., Gamazon, E., Zhang, W., Duan, S., Dolan, M.E., and Cox, N.J. (2010). Trait-associated SNPs are more likely to be eQTLs: annotation to enhance discovery from GWAS. PLoS Genet. *6*, e1000888.
3. Degner, J.F., Pai, A.A., Pique-Regi, R., Veyrieras, J.B., Gaffney, D.J., Pickrell, J.K., De Leon, S., Michelini, K., Lewellen, N., Crawford, G.E., et al. (2012). DNase I sensitivity QTLs are a major determinant of human expression variation. Nature *482*, 390–394.
4. Davis, L.K., Yu, D., Keenan, C.L., Gamazon, E.R., Konkashbaev, A.I., Derks, E.M., Neale, B.M., Yang, J., Lee, S.H., Evans, P., et al. (2013). Partitioning the heritability of Tourette syndrome and obsessive compulsive disorder reveals differences in genetic architecture. PLoS Genet. *9*, e1003864.
5. Gusev, A., Ko, A., Shi, H., Bhatia, G., Chung, W., Penninx, B.W., Jansen, R., de Geus, E.J., Boomsma, D.I., Wright, F.A., et al. (2016). Integrative approaches for large-scale transcriptome-wide association studies. Nat. Genet. *48*, 245–252.
6. Torres, J.M., Gamazon, E.R., Parra, E.J., Below, J.E., Valladares-Salgado, A., Wacher, N., Cruz, M., Hanis, C.L., and Cox, N.J.

(2014). Cross-tissue and tissue-specific eQTLs: partitioning the heritability of a complex trait. Am. J. Hum. Genet. *95*, 521–534.

7. Giambartolomei, C., Vukcevic, D., Schadt, E.E., Franke, L., Hingorani, A.D., Wallace, C., and Plagnol, V. (2014). Bayesian test for colocalisation between pairs of genetic association studies using summary statistics. PLoS Genet. *10*, e1004383.
8. Hormozdiari, F., van de Bunt, M., Segrè, A.V., Li, X., Joo, J.W.J., Bilow, M., Sul, J.H., Sankararaman, S., Pasaniuc, B., and Eskin, E. (2016). Colocalization of GWAS and eQTL Signals Detects Target Genes. Am. J. Hum. Genet. *99*, 1245–1260.
9. Smith, G.D., and Ebrahim, S. (2003). 'Mendelian randomization': can genetic epidemiology contribute to understanding environmental determinants of disease? Int. J. Epidemiol. *32*, 1–22.
10. Freeman, G., Cowling, B.J., and Schooling, C.M. (2013). Power and sample size calculations for Mendelian randomization studies using one genetic instrument. Int. J. Epidemiol. *42*, 1157–1163.
11. Zhu, Z., Zhang, F., Hu, H., Bakshi, A., Robinson, M.R., Powell, J.E., Montgomery, G.W., Goddard, M.E., Wray, N.R., Visscher, P.M., and Yang, J. (2016). Integration of summary data from GWAS and eQTL studies predicts complex trait gene targets. Nat. Genet. *48*, 481–487.
12. Gamazon, E.R., Wheeler, H.E., Shah, K.P., Mozaffari, S.V., Aquino-Michaels, K., Carroll, R.J., Eyler, A.E., Denny, J.C., Nicolae, D.L., Cox, N.J., Im, H.K.; and GTEx Consortium (2015). A gene-based association method for mapping traits using reference transcriptome data. Nat. Genet. *47*, 1091–1098.
13. Mancuso, N., Shi, H., Goddard, P., Kichaev, G., Gusev, A., and Pasaniuc, B. (2017). Integrating Gene Expression with Summary Association Statistics to Identify Genes Associated with 30 Complex Traits. Am. J. Hum. Genet. *100*, 473–487.
14. Barbeira, A.N., Dickinson, S.P., Bonazzola, R., Zheng, J., Wheeler, H.E., Torres, J.M., Torstenson, E.S., Shah, K.P., Garcia, T., Edwards, T.L., et al.; GTEx Consortium (2018). Exploring the phenotypic consequences of tissue specific gene expression variation inferred from GWAS summary statistics. Nat. Commun. *9*, 1825.
15. Wheeler, H.E., Shah, K.P., Brenner, J., Garcia, T., Aquino-Michaels, K., Cox, N.J., Nicolae, D.L., Im, H.K.; and GTEx Consortium (2016). Survey of the Heritability and Sparse Architecture of Gene Expression Traits across Human Tissues. PLoS Genet. *12*, e1006423.
16. Hemani, G., Bowden, J., and Davey Smith, G. (2018). Evaluating the potential role of pleiotropy in Mendelian randomization studies. Hum. Mol. Genet. *27* (R2), R195–R208.
17. Stacey, D., Fauman, E.B., Ziemek, D., Sun, B.B., Harshfield, E.L., Wood, A.M., Butterworth, A.S., Suhre, K., and Paul, D.S. (2019). ProGeM: a framework for the prioritization of candidate causal genes at molecular quantitative trait loci. Nucleic Acids Res. *47*, e3.
18. Kettunen, J., Tukiainen, T., Sarin, A.P., Ortega-Alonso, A., Tikkanen, E., Lyytikäinen, L.P., Kangas, A.J., Soininen, P., Würtz, P., Silander, K., et al. (2012). Genome-wide association study identifies multiple loci influencing human serum metabolite levels. Nat. Genet. *44*, 269–276.
19. Suhre, K., and Gieger, C. (2012). Genetic variation in metabolic phenotypes: study designs and applications. Nat. Rev. Genet. *13*, 759–769.
20. Shin, S.Y., Fauman, E.B., Petersen, A.K., Krumsiek, J., Santos, R., Huang, J., Arnold, M., Erte, I., Forgetta, V., Yang, T.P., et al.; Multiple Tissue Human Expression Resource (MuTHER) Consortium (2014). An atlas of genetic influences on human blood metabolites. Nat. Genet. *46*, 543–550.
21. Kastenmüller, G., Raffler, J., Gieger, C., and Suhre, K. (2015). Genetics of human metabolism: an update. Hum. Mol. Genet. *24* (R1), R93–R101.
22. Battle, A., Brown, C.D., Engelhardt, B.E., Montgomery, S.B.; GTEx Consortium; Laboratory, Data Analysis &Coordinating Center (LDACC)—Analysis Working Group; Statistical Methods groups—Analysis Working Group; Enhancing GTEx (eGTEx) groups; NIH Common Fund; NIH/NCI; NIH/NHGRI; NIH/NIMH; NIH/NIDA; Biospecimen Collection Source Site—NDRI; Biospecimen Collection Source Site—RPCI; Biospecimen Core Resource—VARI; Brain Bank Repository—University of Miami Brain Endowment Bank; Leidos Biomedical—Project Management; ELSI Study; Genome Browser Data Integration &Visualization—EBI; Genome Browser Data Integration &Visualization—UCSC Genomics Institute, University of California Santa Cruz; Lead analysts; Laboratory, Data Analysis &Coordinating Center (LDACC); NIH program management; Biospecimen collection; Pathology; and eQTL manuscript working group (2017). Genetic effects on gene expression across human tissues. Nature *550*, 204–213.
23. Ritchie, M.E., Phipson, B., Wu, D., Hu, Y., Law, C.W., Shi, W., and Smyth, G.K. (2015). limma powers differential expression analyses for RNA-sequencing and microarray studies. Nucleic Acids Res. *43*, e47.
24. Leek, J.T., Johnson, W.E., Parker, H.S., Jaffe, A.E., and Storey, J.D. (2012). The sva package for removing batch effects and other unwanted variation in high-throughput experiments. Bioinformatics *28*, 882–883.
25. Delaneau, O., Ongen, H., Brown, A.A., Fort, A., Panousis, N.I., and Dermitzakis, E.T. (2017). A complete tool set for molecular QTL discovery and analysis. Nat. Commun. *8*, 15452.
26. Suhre, K., Shin, S.Y., Petersen, A.K., Mohney, R.P., Meredith, D., Wägele, B., Altmaier, E., Deloukas, P., Erdmann, J., Grundberg, E., et al.; CARDIoGRAM (2011). Human metabolic individuality in biomedical and pharmaceutical research. Nature *477*, 54–60.
27. Friedman, J., Hastie, T., and Tibshirani, R. (2010). Regularization Paths for Generalized Linear Models via Coordinate Descent. J. Stat. Softw. *33*, 1–22.
28. Yang, J., Benyamin, B., McEvoy, B.P., Gordon, S., Henders, A.K., Nyholt, D.R., Madden, P.A., Heath, A.C., Martin, N.G., Montgomery, G.W., et al. (2010). Common SNPs explain a large proportion of the heritability for human height. Nat. Genet. *42*, 565–569.
29. Wishart, D.S., Feunang, Y.D., Marcu, A., Guo, A.C., Liang, K., Vázquez-Fresno, R., Sajed, T., Johnson, D., Li, C., Karu, N., et al. (2018). HMDB 4.0: the human metabolome database for 2018. Nucleic Acids Res. *46* (D1), D608–D617.
30. Burgess, S., Butterworth, A., and Thompson, S.G. (2013). Mendelian randomization analysis with multiple genetic variants using summarized data. Genet. Epidemiol. *37*, 658–665.
31. Mikhaylova, A.V., and Thornton, T.A. (2019). Accuracy of Gene Expression Prediction From Genotype Data With PrediXcan Varies Across and Within Continental Populations. Front. Genet. *10*, 261.
32. Mogil, L.S., Andaleon, A., Badalamenti, A., Dickinson, S.P., Guo, X., Rotter, J.I., Johnson, W.C., Im, H.K., Liu, Y., and Wheeler, H.E. (2018). Genetic architecture of gene expression traits across diverse populations. PLoS Genet. *14*, e1007586.

33. Barfield, R., Feng, H., Gusev, A., Wu, L., Zheng, W., Pasaniuc, B., and Kraft, P. (2018). Transcriptome-wide association studies accounting for colocalization using Egger regression. Genet. Epidemiol. *42*, 418–433.
34. Mancuso, N., Freund, M.K., Johnson, R., Shi, H., Kichaev, G., Gusev, A., and Pasaniuc, B. (2019). Probabilistic fine-mapping of transcriptome-wide association studies. Nat. Genet. *51*, 675–682.
35. Lenich, A.C., and Goodman, S.I. (1986). The purification and characterization of glutaryl-coenzyme A dehydrogenase from porcine and human liver. J. Biol. Chem. *261*, 4090–4096.
36. Waldmann, P., Mészáros, G., Gredler, B., Fuerst, C., and Sölkner, J. (2013). Evaluation of the lasso and the elastic net in genome-wide association studies. Front. Genet. *4*, 270.
37. Lloyd-Jones, L.R., Holloway, A., McRae, A., Yang, J., Small, K., Zhao, J., Zeng, B., Bakshi, A., Metspalu, A., Dermitzakis, M., et al. (2017). The Genetic Architecture of Gene Expression in Peripheral Blood. Am. J. Hum. Genet. *100*, 228–237.
38. Gamazon, E.R., Segrè, A.V., van de Bunt, M., Wen, X., Xi, H.S., Hormozdiari, F., Ongen, H., Konkashbaev, A., Derks, E.M., Aguet, F., et al.; GTEx Consortium (2018). Using an atlas of gene regulation across 44 human tissues to inform complex disease- and trait-associated variation. Nat. Genet. *50*, 956–967.
39. Wainberg, M., Sinnott-Armstrong, N., Mancuso, N., Barbeira, A.N., Knowles, D.A., Golan, D., Ermel, R., Ruusalepp, A., Quertermous, T., Hao, K., et al. (2019). Opportunities and challenges for transcriptome-wide association studies. Nat. Genet. *51*, 592–599.
40. Wu, L., Candille, S.I., Choi, Y., Xie, D., Jiang, L., Li-Pook-Than, J., Tang, H., and Snyder, M. (2013). Variation and genetic control of protein abundance in humans. Nature *499*, 79–82.

The American Journal of Human Genetics, Volume *106*

Supplemental Data

A Multi-tissue Transcriptome Analysis of Human Metabolites Guides Interpretability of Associations Based on Multi-SNP Models for Gene Expression

Anne Ndungu, Anthony Payne, Jason M. Torres, Martijn van de Bunt, and Mark I. McCarthy

SUPPLEMENTAL FIGURES

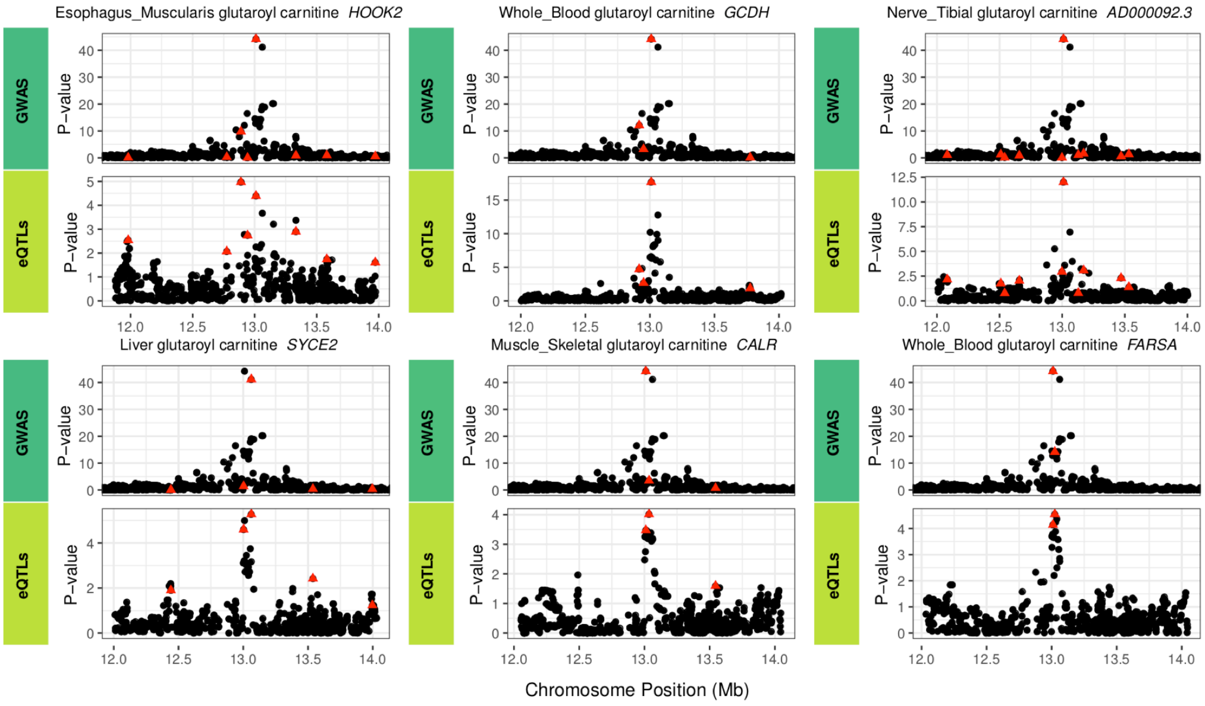

Figure S1. Gene expression co-regulation at the *GCDH* locus. Six genes at the *GCDH* locus whose predicted expression significantly mediated glutarylcarnitine levels. The multi-SNP models for all six genes included the *cis*-eQTL rs8012 that regulates the expression for all the genes. *GCDH* is the true causal gene influencing metabolite levels at this locus.

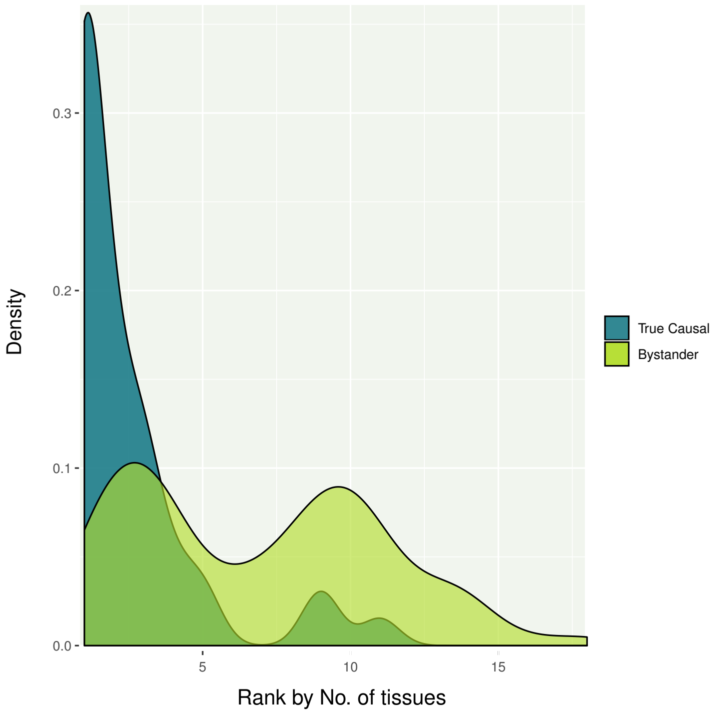

Figure S2. Causal and bystander gene ranks by number of tissues. Comparison of ranks by tissue counts for true causal genes and bystander genes at each locus. True causal genes ranked higher (significant in a more tissues) than bystander genes at the same locus.
